# Qingqiao polyphenols may alleviate multi-organ inflammatory injury caused by *Escherichia coli* infection in chickens by regulating pyroptosis in a gut microbiota-independent manner

**DOI:** 10.3389/fvets.2026.1976802

**Published:** 2026-10-29

**Authors:** Limin Chao, Yuchao Feng, Yangming Liang, Wen Li, Yating Yu, Yan Yu, Pei Gao, Jinyou Ma

**Affiliations:** 1 College of Animal Science and Veterinary Medicine, Henan Institute of Science and Technology, Xinxiang, China; 2 Henan International Joint Laboratory of Animal Health Breeding and Disease Prevention and Control, Xinxiang, Henan, China

**Keywords:** chick, *Escherichia coli*, multi-organ inflammatory injury, pyroptosis, Qingqiao polyphenols

## Abstract

**Background:**

This study systematically elucidates the mechanism by which Qingqiao polyphenols (QP) alleviate bacterial infectious lesions in poultry by regulating the pyroptosis signaling pathway.

**Methods:**

An *Escherichia coli* (*E. coli*) O157 infection model was established and evaluate the effect of QP on infected chickens.

**Results:**

Results showed QP significantly alleviated infection-induced systemic damage, manifested as improved pathological lesions in liver and lungs, and restored mucosal structures in duodenum, jejunum, and ileum. Further studies demonstrated that QP alleviates inflammatory damage in the liver, lungs, and intestines (duodenum, jejunum, ileum) of chickens induced by *E. coli*, by suppressing the pyroptosis signaling pathway. After gut microbial depletion by antibiotics, a lipopolysaccharide (LPS)-induced chicken enteritis model was used to evaluate whether QP can exert anti-inflammatory effects by inhibiting the pyroptosis pathway. The results demonstrated that QP effectively suppressed pyroptosis and alleviated LPS-induced intestinal inflammation, maintaining efficacy even under antibiotic depleted microbiota group conditions. This suggests that the anti-inflammatory effect of QP may be mediated by a pyroptosis pathway that is independent of the gut microbiota.

**Conclusion:**

This study confirms that QP exerts multi-organ protective effects against *E. coli*-induced inflammatory injury via a gut microbiota-independent pyroptosis-inhibitory mechanism, which provides a new theoretical basis for the application of QP as an antibiotic substitute in poultry bacterial disease prevention and control.

## 1 Introduction

Bacterial infectious diseases in poultry represent a significant bottleneck constraining the healthy development of the poultry farming industry, with enteritis and systemic lesions caused by *Escherichia coli* (*E. coli*) infections being particularly prominent (1, 2). *E. coli* is a major pathogenic serotype reported globally and one of the primary pathogens causing colibacillosis in chicks (3). Lipopolysaccharide (LPS) constitutes the primary component of the outer membrane in the cell wall of Gram-negative *E. coli*. It serves as a key virulence factor and the primary pathogenic substance responsible for inducing severe inflammatory responses and infections in hosts (4). *E. coli* disease poses a dual threat to the poultry industry and public health due to its contribution to high mortality rates in poultry worldwide, substantial economic losses, and potential zoonotic risks (5, 6).

In the pathological process of bacterial infection, pyroptosis is a major programmed cell death that significantly exacerbates tissue damage (7, 8). Upon recognition of pathogen-associated molecular patterns (PAMPs), NLRP3 inflammasomes assemble with the adaptor protein ASC to form a complex, which subsequently recruits and activates Caspase-1 (9). Activated Caspase-1 drives inflammation through two distinct pathways. First, it cleaves Gasdermin D (GSDMD), leading to the formation of pores in the cell membrane and ultimately inducing pyroptosis. Second, it promotes the maturation and secretion of proinflammatory cytokines including IL-1β and IL-18 (10, 11). This process further amplifies the inflammatory cascade, leading to dysfunction at the site of infection and in distant organs. Recent studies indicate that abnormal activation of pyroptosis is closely associated with various inflammatory diseases, suggesting this pathway may represent a critical target for intervening in infectious injury (7, 12).

The overuse of antibiotics has exacerbated the growing threat of drug resistance, creating an urgent demand for new veterinary drugs in livestock and poultry farming (13, 14). Chinese herbal medicines derived from natural sources have garnered considerable attention from researchers due to their multi-target effects, low potential for drug resistance, and other advantageous properties. *Forsythia suspensa*, a traditional Chinese medicinal herb, yields fruits classified as “Qingqiao” (immature, green) and “Laoqiao” (fully ripe, yellow) based on harvest timing. Traditional Chinese medicine clinical practice holds that Qingqiao possesses superior medicinal value compared to Laoqiao (15). Qingqiao polyphenols (QP) are plant polyphenols extracted from dried green forsythia fruits. They contain multiple active components such as Forsythoside A, Phillyrin, and Phillygenin, and have been shown to exhibit a range of pharmacological effects including anti-inflammatory, antioxidant, and antibacterial properties (16–18).

Our initial findings showed that a forsythia extract rich in polyphenols ameliorated ulcerative colitis in mice via the Nrf2-NLRP3 signaling axis (19). Further studies demonstrated that its active component QP specifically inhibits NLRP3/AIM2 inflammasome-mediated pyroptosis, thereby effectively improving disease progression (18). However, despite QP’s demonstrated anti-inflammatory and antioxidant activity in mammalian models, its protective effects and mechanisms against bacterial infections in poultry remain understudied. Given its natural origin and multifaceted pharmacological properties, QP holds significant potential as an alternative to antibiotics for treating bacterial infections in poultry. This study explores QP’s effects and mechanisms in mitigating systemic damage caused by *E. coli* infection from a pyroptosis perspective, providing theoretical support for developing novel anti-bacterial infection agents for poultry.

## 2 Materials and methods

### 2.1 Materials and reagents

RIPA lysis buffer, nutrient agar medium, phenylmethylsulfonyl fluoride (PMSF), Luria-Bertani (LB) medium, and eosin-methylene blue differential agar medium were purchased from Beijing Solarbio Science & Technology Co., Ltd. (Beijing, China). BCA protein concentration assay kit was purchased from Shanghai Biyuntian Biotechnology Institute (Shanghai, China). Trizol reagent and PrimeScript™ RT reverse transcription kit were purchased from Beijing Baoruiyi Biotechnology Co., Ltd. (Beijing, China). The lactate dehydrogenase (LDH) assay kit and myeloperoxidase (MPO) assay kit were both procured from Wuhan Eritai Biotechnology Co., Ltd. (Wuhan, China). Assay kits for total superoxide dismutase (SOD) activity and malondialdehyde (MDA) content were obtained from Nanjing Jiancheng Bioengineering Institute based in Nanjing, P. R. China. Hefei Bomei Biotechnology Co., Ltd. (Hefei, China) supplied the antibiotics used in this study, namely ampicillin, neomycin, metronidazole, streptomycin and vancomycin. Enrofloxacin hydrochloride soluble powder was procured from Wuhan Huisheng Biotechnology Co., Ltd. located in Wuhan, China. For LPS, we purchased it from Sigma-Aldrich in St. Louis, Missouri, United States.

### 2.2 Extraction, purification, and content determination of polyphenols

QP was sourced from Yuzheng Traditional Chinese Medicine Tablets Co., Ltd., which is located in Anguo City, Hebei Province, China. The QP was pulverized using a traditional Chinese medicine grinder and sieved through a 40-mesh screen. A crude extract was obtained via ultrasonic-assisted extraction (53 °C, 0.72 h, 62% ethanol, solid–liquid ratio 38 mL/g). This extract underwent adsorption on D101 macroporous resin (solid–liquid ratio 1:5, 25 °C, 180 rpm, 24 h) and desorption with 60% ethanol, followed by freeze-drying, yielding polyphenol powder. Polyphenol content was determined using the Folin–Ciocalteu method. The absorbance value of the sample at 760 nm was substituted into the gallic acid standard curve for calculation, ultimately yielding the extraction rate of QP.

### 2.3 Bacterial strain

The *E. coli* O157: H7strain used in this experiment was preserved and provided by Henan Institute of Science and Technology (HIST157). The preserved frozen strain was inoculated into 5 mL LB liquid medium, followed by 12 h of shaking culture at 37 °C and 180 rpm. The bacterial suspension was inoculated onto LB solid medium via the four-quadrant streaking technique. Following 12 h of incubation at 37 °C, a single colony was selected and transferred into 5 mL of fresh LB liquid medium. This was cultured under identical conditions (37 °C, 180 rpm) for 12 h. Finally, the McFarland turbidity method was employed. For subsequent testing purposes, the bacterial suspension was standardized to a concentration of 5 × 10^8^ CFU/mL using sterile PBS.

### 2.4 Animals and experimental design

Day-old Jinghong laying hens (30.85 ± 0.71 g) were purchased from the Datian Hatchery in Xinxiang County, China. The chickens were acclimated for 3 days to the experimental conditions prior to the study. For the experiment, chickens were housed under controlled conditions: constant temperature at 30 ± 2 °C, relative humidity ranging from 50 to 60%, and a 12-h light–dark cycle. Each experimental group was allocated 3 independent identical cages, with 5–6 chicks per cage, resulting in a total of 18 cages across 6 experimental groups. The stocking density was strictly controlled at 6 chicks per square meter, which conforms to the standard welfare specification for broiler rearing during the challenge period. They had free access to standard laboratory diet and fresh drinking water. The protocol was approved by the Animal Experiment Ethics Review Committee of Henan Institute of Science and Technology (Approval No: LLSC2025069). All operations strictly complied with the Guidelines for the Welfare and Ethics of Laboratory Animals to ensure minimal distress to the animals.

(1) Ninety 4-day-old healthy chicks with similar baseline body weight were individually tagged, sorted by body weight, and divided into 15 consecutive weight-matched blocks (6 chicks per block). Within each block, chicks were blindly allocated to the 6 experimental groups using pre-shuffled sealed envelopes, forming 15 fully balanced randomization units. Ninety chicks were randomly assigned to 6 groups (*n* = 15): blank control, *E. coli* O157 model, 0.4 g/kg QP, 0.8 g/kg QP, 1.6 g/kg QP, and enrofloxacin positive control (18). From Day 4 to Day 15, QP groups received corresponding QP-supplemented diet, while control and model groups received basal diet. After the Day 9 challenge, the positive control group was given 0.367 g/L enrofloxacin in drinking water for 5 consecutive days. All groups except the blank control received a single intraperitoneal injection of 0.2 mL *E. coli* O157 suspension (5 × 10^8^ CFU/mL, 1 × 10^8^ CFU/chick) on Day 9, while the control group received equal volume of sterile saline. Clinical symptoms were recorded daily. On day 15, all feed was removed from each cage, and drinking water was provided ad libitum throughout the fasting period. After 24 h fasting on Day 15, all chicks were euthanized on Day 16, and heart, liver, spleen, lung, intestinal segments and immune organs were collected for subsequent analysis.
(2) Sixty 4-day-old chicks were randomly assigned to 4 groups (*n* = 15): normal control, LPS model, QP treatment (intact microbiota), and QP treatment (near-depleted microbiota). From Day 4 to Day 8, the control, LPS model and microbiota-depleted groups were treated with broad-spectrum antibiotic cocktail (1.0 g/L ampicillin, 1.0 g/L streptomycin, 1.0 g/L metronidazole, 0.5 g/L vancomycin) in drinking water for 5 days to deplete gut commensal bacteria (20), while the other groups received normal drinking water. All QP-treated groups were fed diet containing 1.6 g/kg QP from Day 4 to Day 15. On Day 9, 11 and 13, LPS challenge groups received intraperitoneal injection of 1 mg/kg LPS per body weight, while the control group received equivalent saline. All chicks were sampled after 24 h fasting on Day 15, with ileum, liver, spleen and immune organs collected for detection.

### 2.5 *In vitro* experiments

This study employed the pre-inoculated pour plate method and broth microdilution technique to systematically evaluate the *in vitro* antibacterial activity of QP (at a concentration of 175 mg/mL) (21). In brief, the pre-inoculated pour plate method involved mixing *E. coli* O157 suspension with molten agar and pouring it onto plates (final concentration 5 × 10^5^ CFU/mL). After solidification, sterile punches (6 mm diameter) were used to create wells. QP solution, enrofloxacin (positive control), and physiological saline (negative control) were added to the wells, respectively. Incubate the cultures at 37 °C for 18–24 h, after which the diameter of each inhibition zone should be measured. This entire experimental process was replicated independently on three separate occasions.

MIC/MBC Determination by Broth Dilution Method: In a 96-well plate, perform two-fold serial dilution of the drug using LB broth as the diluent. Then, add an equal volume of bacterial suspension to every well, ensuring the final bacterial density reaches 5 × 10^6^ CFU/mL. Set up negative controls containing LB broth and the test sample, while positive controls contain only LB broth and bacterial suspension. Following a 24-h incubation at 37 °C, the lowest drug concentration at which no visible bacterial growth was detected was documented as the MIC. For MBC determination, 100 μL of culture medium from wells containing MIC and higher concentrations was spread onto LB agar plates. The minimum concentration showing no bacterial growth after 24-h incubation at 37 °C was recorded as the MBC.

### 2.6 Determination of bacterial load

A small piece of tissue was cut aseptically and weighed in a homogenization tube to obtain its wet weight. All samples were temporarily stored at 4 °C and processed uniformly after collection. During processing, 1 mL of sterile PBS, one 5-mm sterile steel ball, and one 3-mm sterile steel ball were added to each tube. The tubes were placed in a pre-cooled tissue grinder at 4 °C and homogenized thoroughly to prepare tissue homogenates. Subsequently, under aseptic conditions in a clean bench, 100 μL of tissue homogenate was taken and serially diluted 10-fold with sterile PBS. Three suitable dilutions were selected; 100 μL of each dilution was pipetted and spread evenly onto Eosin Methylene Blue (EMB) agar plates. The plates were inverted and incubated overnight at 37 °C. After incubation, colonies with typical characteristics of *E. coli* O157 (with a dark purple or black center and a metallic luster) were counted on each plate. Plates with 30–300 colonies were selected for counting. The *E. coli* load per gram of tissue wet weight (CFU/g) was calculated based on the dilution factors and expressed as log10 CFU/g to evaluate colonization by pathogenic bacteria at different concentrations in ileal tissue.

### 2.7 Body weight and organ index

During the experiment, the body weights of chicks in each group were measured on an empty stomach at Day 1 (experiment start), Day 4 (pre-intervention), and Day 9 (pre-challenge). At the conclusion of the experiment, each group of chickens was weighed, and organ tissues including the heart, liver, spleen, lungs, bursa of Fabricius, and thymus were collected. Organ indices were calculated post-weighing using the formula: Organ Index = Organ Weight (g)/Body Weight (kg) (22).

### 2.8 Determination of bacterial load in organs

Following homogenization of infected tissues, a 10-fold serial dilution combined with plate-count culture technique was employed to quantitatively determine the number of viable pathogens per unit mass of tissue, thereby assessing pathogen colonization in various target organs (23).

### 2.9 Histological analysis

Tissue specimens were immersed in 4% paraformaldehyde, processed through a graded ethanol series for dehydration, and embedded in paraffin following standard procedures. Serial sections were cut with a microtome, stained with hematoxylin and eosin (HE), and examined under light microscopy to acquire pathological images. According to the scoring criteria in Supplementary Table S1, two pathologists participated in the scoring. Prior to the formal evaluation, they completed 10% uniform calibration training in all randomly selected slices to ensure that the inter observer correlation coefficient of the scoring results was greater than 0.92, meeting the reproducibility criteria. All investigators performing laboratory analyses, histological scoring and data analysis were fully blinded to treatment group assignments throughout the entire experimental process.

### 2.10 Serum related indicators

Collect chicken blood using coagulation vessels, place the coagulation vessels at 37 °C for 2 h, and centrifuge at low speed to separate the serum. Measure the following serum parameters using commercial kits: MDA content (Kit No.: A003-1), SOD activity (A001-3), MPO content (E-BC-K074-M), LDH activity (E-BC-K046-M). All experiments strictly followed the manufacturers’ protocols and instructions.

### 2.11 RNA extraction and quantitative real-time PCR

Following the manufacturer’s instructions, Trizol reagent was used to isolate total RNA from tissue samples. RNA purity (A260/A280 = 1.9–2.1) and integrity were verified, and eligible samples were reverse-transcribed into cDNA for subsequent qRT-PCR assays. The 20 μL qRT-PCR system contained 2 × SYBR Green Master Mix, 10 μM paired primers, diluted cDNA template and nuclease-free water. Thermal cycling conditions were: 95 °C for 3 min; 40 cycles of 95 °C for 5 s, 60 °C for 30 s, followed by melting curve analysis from 65 °C to 95 °C to confirm amplification specificity. Subsequently, qRT-PCR was employed to detect the relative expression levels of pyroptosis-related genes (NLRP3, Caspase-1, GSDMD, ASC, TNF-α, IL-18, and IL-1β) in the tissues. Primers were purchased from Shanghai Sangon Biotech Co., Ltd., as listed in Table 1.

**Table 1:** Sequences of primers for qRT-PCR.

Gene	Direction	Gene sequence
*β-actin*	Forward primer	CTGACTGACCGCGTTACTCC
	Reverse primer	CATACCAACCATCACACCCTGA
*NLRP3*	Forward primer	CGACGAGGAGACAGGTTTGTT
	Reverse primer	ACAGGTCTGCTTCCACTTTCC
*GSDMD*	Forward primer	CTGCTCTGATCTACAGGTTGTTCCG
	Reverse primer	GCTCAGTGCCAAGGTGCCATC
*Caspase-1*	Forward primer	CAAGAGTAATGGGACCACGGACATC
	Reverse primer	CACGGCAGCACTGGATAATGACC
*ASC*	Forward primer	CCAGCAAAGCCTCCGCCAAG
	Reverse primer	CAGCCACCTCATACGCCTTCTTC
*IL-1β*	Forward primer	CACTGGGCATCAAGGGCTACAAG
	Reverse primer	GTCCAGGCGGTAGAAGATGAAGC
*IL-18*	Forward primer	TGATGAGCTGGAATGCGATGCC
	Reverse primer	TGGACGAACCACAAGCAACTGG
*TNF-α*	Forward primer	TGATCGTGACACGTCTCTGC
	Reverse primer	CAACCAGCTATGCACCCCAG

### 2.12 Western blot analysis

Jejunal tissue was homogenized in ice-cold RIPA lysis buffer (with 1% protease inhibitor), lysed for 30 min, and centrifuged at 12,000 × g (4 °C, 15 min). Total protein concentration was quantified via BCA assay, then adjusted to uniform concentration for SDS-PAGE separation. Separated proteins were wet-transferred onto Immobilon-PSQ PVDF membranes, blocked with 5% non-fat milk for 2 h, and incubated with primary antibodies at 4 °C for ≥12 h. Antibodies and dilutions used were: NLRP3 (1:1,500, Abmart, P60622R3), Caspase-1 p20 (1:1,500, Abmart, PC6368), GSDMD (1:1,000, Solarbio, K009328P), IL-1β (1:1,500, Beyotime, AF7209), ASC (1:2,000, Beyotime, AF6234), and β-actin (1:3,000, Beyotime, AF5003). Following incubation, the membranes were rinsed six times with PBST and then incubated for 1 h with a horseradish peroxidase-conjugated secondary antibody (1:10,000, Beyotime, A0208). Protein bands were detected using a high-sensitivity chemiluminescent substrate, and their intensities were quantified with ImageJ software (ImageJ 1.44p).

### 2.13 Statistical analysis

Statistical analysis was performed using SPSS 20.0 (IBM, Armonk, NY, United States), and graphs were created with GraphPad Prism 5.0 (GraphPad, San Diego, CA, United States). Results are expressed as mean ± standard error of the mean (SEM). Comparisons among groups were carried out using one-way analysis of variance (ANOVA) followed by Tukey’s HSD test. A significance threshold of *p* < 0.05 was adopted.

## 3 Results

### 3.1 Principal component analysis of QP

The QP extract used in this study is from the same batch as that in the previous research (18). Its polyphenol extraction rate was determined by the Folin–Ciocalteu method to be 36.53% (test conditions: wavelength 760 nm, calculated using the standard curve equation *c* = 0.0199x – 0.0029). Furthermore, as described in our published literature, the high-performance liquid chromatography (HPLC) analysis of the three major active components in this extract yielded the following results: Forsythoside A at 37.88%, Phillyrin at 3.94%, and Phillygenin at 0.31% (18).

### 3.2 *In vitro* antibacterial activity of QP against *E. coli*

This study employed the pre-inoculated pour plate method and broth microdilution technique to systematically evaluate the *in vitro* antibacterial activity of QP (at a concentration of 175 mg/mL) against *E. coli*. Results showed that QP at 175 mg/mL formed a 13 mm diameter zone of inhibition, while the positive control enrofloxacin produced a 28 mm zone, indicating QP possesses antibacterial activity (Supplementary Figure S1A). Further determination of the MIC and MBC via broth microdilution revealed that bacterial broth clarity progressively increased with rising QP concentrations (becoming markedly clear from the second well onward). With both MIC and MBC values against *E. coli* reaching 43.75 mg/mL. No bacterial colonies were observed on agar plates inoculated with dilutions at or above the MIC concentration (Supplementary Figure S1B). In summary, QP exhibit both bacteriostatic and bactericidal activity against *E. coli in vitro*.

### 3.3 Effects of QP on body weight, organ index, and bacterial load in organs of *E. coli*-infected chicks

To investigate the effects of QP on body weight, organ indices, and bacterial load in organs of *E. coli*-infected chicks, thereby clarifying its intervention role in infection-induced systemic damage. Results showed that *E. coli* infection significantly inhibited chick weight gain (*p* < 0.05) (Figure 1A), leading to significantly elevated organ indices in the liver, spleen, heart, and lungs (*p* < 0.05), while thymus and bursa of Fabricius indices were markedly reduced (*p* < 0.05) (Table 2). QP intervention significantly alleviated these tissue injuries and immune organ atrophy (*p* < 0.05). Plate-counting analysis of bacterial loads in organs revealed significantly elevated *E. coli* colonization in the liver, spleen, lungs, and intestines of infected chicks. Following QP treatment, bacterial loads in all organs were significantly reduced (*p* < 0.05), indicating its efficacy in inhibiting *E. coli* migration and colonization within visceral tissues (Figure 1B). Compared to the other QP treatment groups, the H-dose QP group showed the highest weight gain in chickens, and the bacterial load in the liver, spleen, heart, and lungs was also lower. Compared with post-infection enrofloxacin, prophylactic H-dose QP pretreatment showed the strongest protective effect against *E. coli*–induced systemic tissue damage, comparable to the positive control drug enrofloxacin. In summary, QP alleviates pathological damage caused by *E. coli* infection by regulating organ indices, mitigating immune organ damage, and reducing bacterial load in organs.

**Figure 1:**
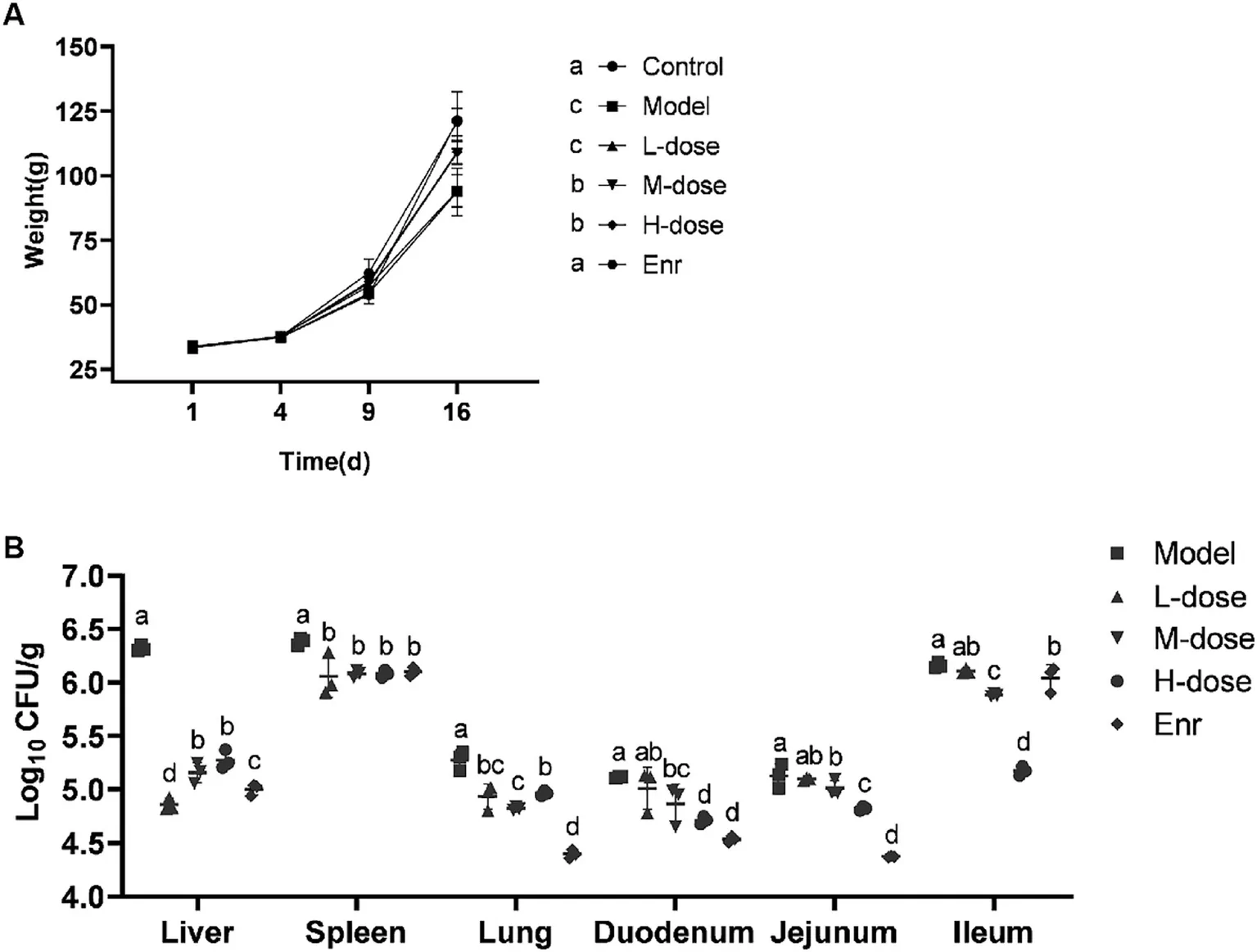
Effects of QP on body weight and bacterial load in organs of *E. coli*-infected chicks. **(A)** Mean body weight changes in each group (*n* = 8). **(B)** Comparison of bacterial load in various tissues and organs. Data are from at least three independent replicates, with results expressed as mean ± SEM. The use of distinct lowercase letters marks significant between‑group differences (*p* < 0.05).

**Table 2:** Organ index analysis of *E. coli*-infected broilers.

Items (%)	Treatments						SEM	*p*-value
	Control	Model	L-dose	M-dose	H-dose	Enr		
Liver coefficient	25.23^cd^	34.72^a^	32.18^b^	26.50^c^	25.96^cd^	24.79^d^	0.701	0.001
Splenic coefficient	0.85^d^	6.55^a^	2.17^b^	1.22^c^	1.06^cd^	0.99^cd^	0.128	0.001
Heart coefficient	6.91^d^	11.87^a^	9.75^b^	9.12^c^	7.02^d^	6.97^d^	0.283	0.001
Lung coefficient	5.86^c^	8.64^a^	7.83^a^	6.88^b^	6.80^b^	6.48^bc^	0.409	0.001
Bursae coefficient of Fabricius	3.73^ab^	2.29^d^	2.89^cd^	3.12^bc^	3.73^ab^	3.89^a^	0.308	0.001
Thymus coefficient	5.01^a^	3.76^c^	3.93^bc^	4.55^ab^	4.73^ab^	4.57^ab^	0.417	0.034

Organ Index = Organ Weight (g)/Body Weight (kg). The use of distinct lowercase letters marks significant between-group differences (*p* < 0.05).

### 3.4 Effects of QP on oxidative stress, MPO levels, and LDH release in *E. coli*-infected chicks

To evaluate the alleviating efficacy of QP on systemic oxidative stress, inflammation, and cellular damage in *E. coli*-infected chicks, we further examined serum oxidative stress markers, MPO levels, and LDH activity. *E. coli* infection significantly increased serum MDA levels and decreased SOD activity in chicks compared with the control group. Compared with the model group, QP treatment significantly lowered the abnormally elevated serum MDA level (*p* < 0.05) and restored SOD activity (Figures 2A,C). The prophylactic H-dose QP pretreatment group exhibited the most pronounced improvement, comparable to the positive control drug enrofloxacin, indicating that QP effectively alleviates oxidative stress induced by *E. coli* infection. Additionally, QP treatment markedly suppressed serum MPO levels, reflecting its capacity to mitigate inflammatory responses (Figure 2B). Furthermore, QP significantly reduced the increase in serum LDH activity (Figure 2D).

**Figure 2:**
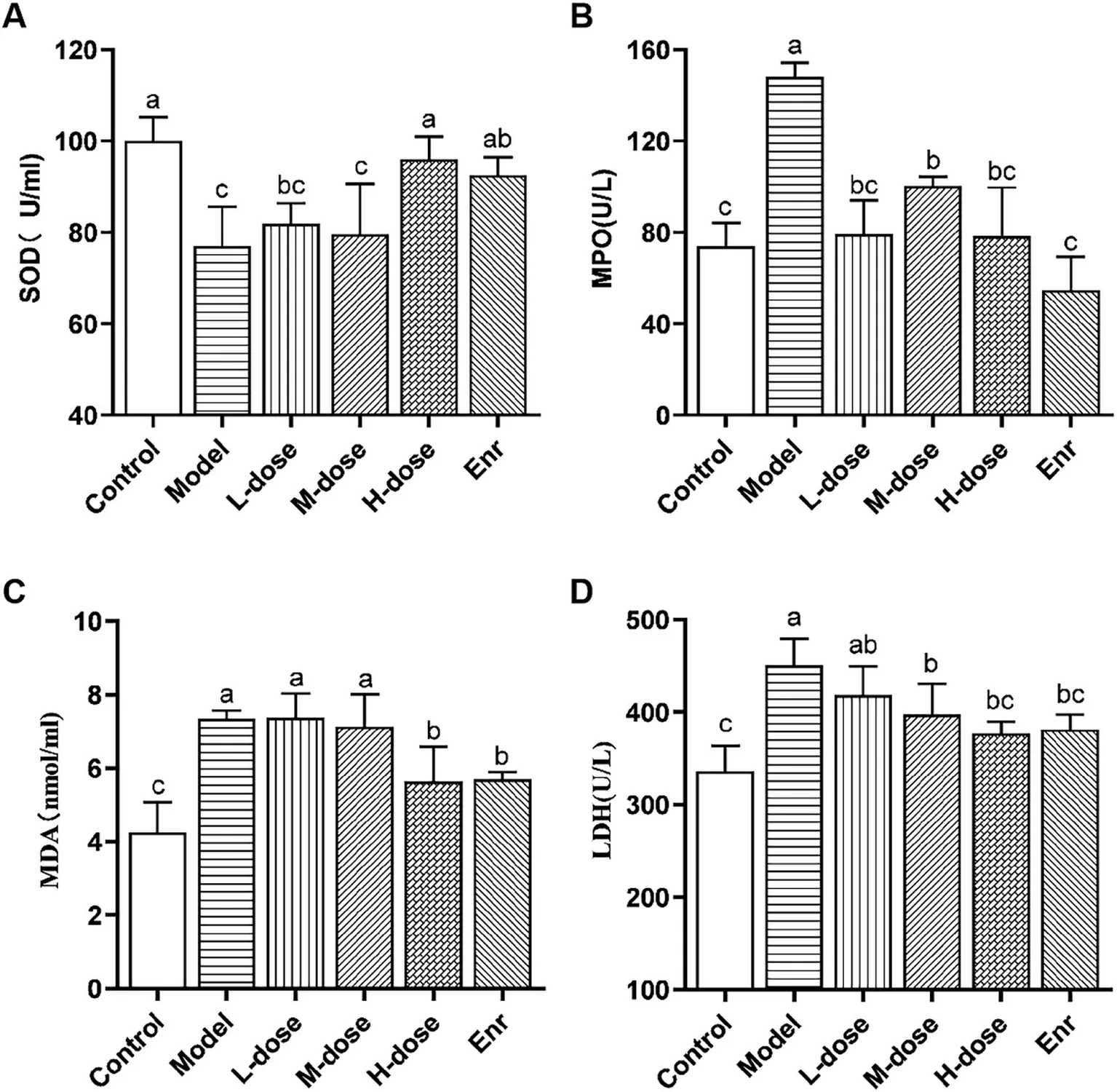
Effects of QP on oxidative stress, MPO levels, and LDH release in *E. coli*-infected chicks. **(A)** Serum SOD (U/mL) activity. **(B)** Serum MPO (U/L) content. **(C)** Serum MDA (nmol/mL) content. **(D)** Serum LDH (U/L) release levels. Data are from at least three independent replicates and are expressed as mean ± SEM. The use of distinct lowercase letters marks significant between‑group differences (*p* < 0.05).

The effect of QP on improving the pathological morphology of organs in *E. coli*-infected chicks.

The therapeutic effect of QP on improving histopathological damage in the liver and lung tissues of *E. coli*-infected chicks was observed through HE staining. Results showed that the liver and lung tissues of chicks in the control group exhibited intact structures with no significant pathological changes. Infected chicks exhibited significant organ damage: the liver showed disrupted hepatic cord arrangement, cellular swelling, reduced hepatic sinusoidal spaces, accompanied by extensive congestion and inflammatory cell infiltration (Figure 3A); lung tissue revealed destruction of pulmonary lobule structure, disappearance of interstitial spaces, narrowing of alveolar spaces, and massive inflammatory cell infiltration (Figure 3B). In the medium-dose and high-dose groups, QP treatment markedly alleviated histopathological lesions in the liver and lungs (Figure 3C). The high-dose QP group demonstrated the most pronounced effect, with tissue morphology resembling that of the control group. The above findings indicate that QP can effectively protect the morphological structure of the liver and lungs in *E. coli*-infected chicks by alleviating tissue inflammation damage.

**Figure 3:**
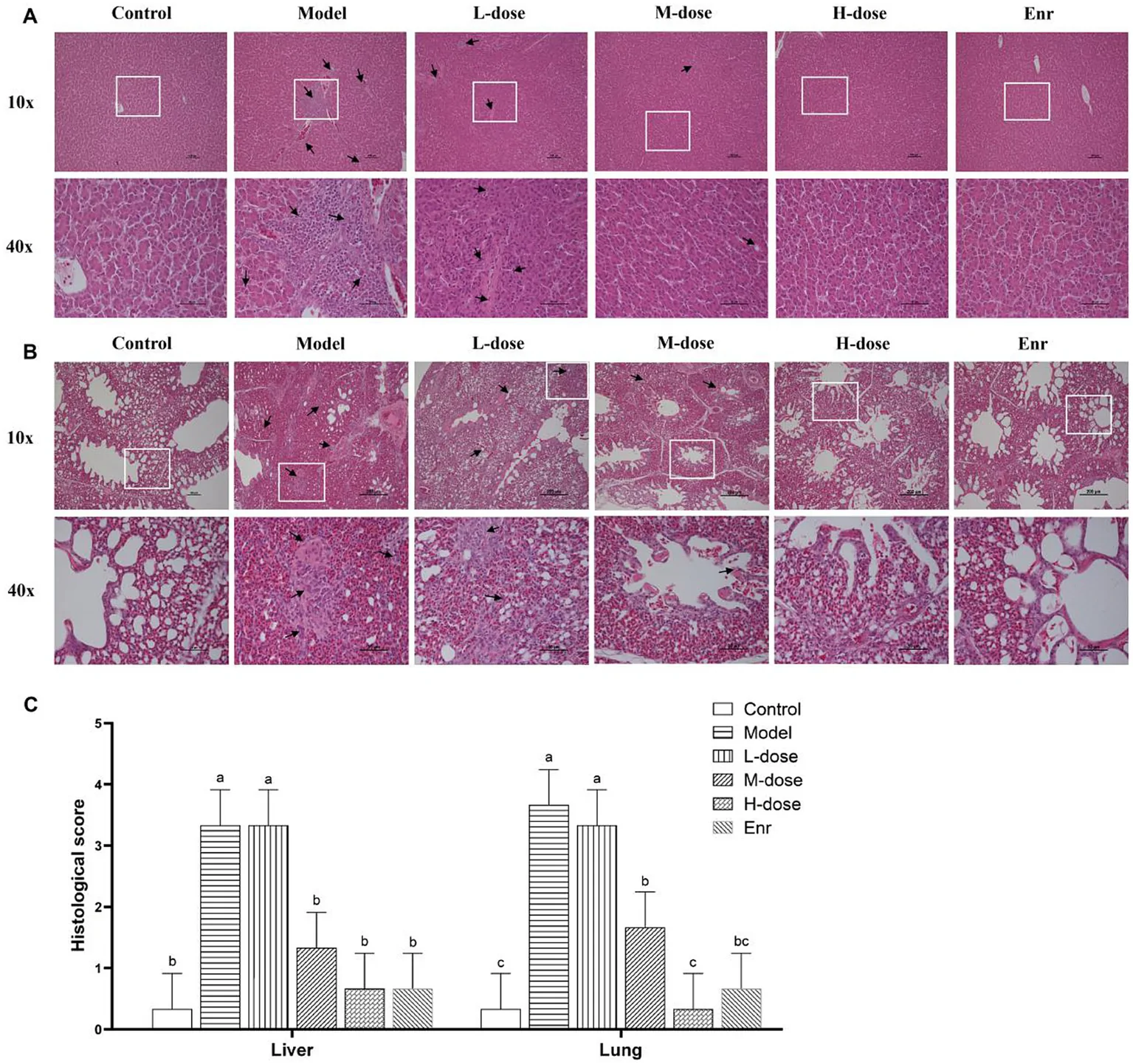
Improvement of pathological morphology in organs of *E. coli*-infected chicks by QP. **(A)** Pathological changes in liver tissue (arrows indicate congestion and inflammatory cell infiltration) and **(B)** pathological changes in lung tissue (arrows indicate destruction of the pulmonary lobular architecture and inflammatory cell infiltration). Hematoxylin and eosin (HE) staining; original magnification: ×100 and ×400. **(C)** Histological score of liver and lung sections. Data are from at least three independent replicates and are expressed as mean ± SEM. The use of distinct lowercase letters marks significant between‑group differences (*p* < 0.05).

### 3.5 The repairing effect of QP on the histopathology of intestinal tissue in *E. coli*-infected chicks

The effect of QP on improving histopathological damage in the intestinal tissues (duodenum, jejunum, and ileum) of *E. coli*-infected chicks was evaluated via HE staining. As shown in Figure 4, the intestinal segments of chicks in the control group exhibited intact structures, neatly arranged villi, and clearly defined mucosal layers. In the model group, extensive pathological damage was observed in the duodenum, jejunum, and ileum, characterized by disordered, fractured, or even detached villi, disrupted mucosal layer structure, significantly thickened mucus layer, massive inflammatory cell infiltration, fragmented vascular structures, and abnormal epithelial cell hyperplasia. After QP intervention, the degree of intestinal injury was reduced to varying degrees in each treatment group, and the intestinal villus structure gradually recovered, mucosal integrity improved, and inflammatory infiltration decreased (Figures 4A–C). The prophylactic H-dose QP pretreatment group and the enrofloxacin positive control group demonstrated significant effects, exhibiting intestinal tissue morphology similar to the control group with no significant pathological alterations (Figure 4D). These findings indicate that QP significantly alleviates intestinal inflammatory damage induced by *E. coli* infection in chicks.

**Figure 4:**
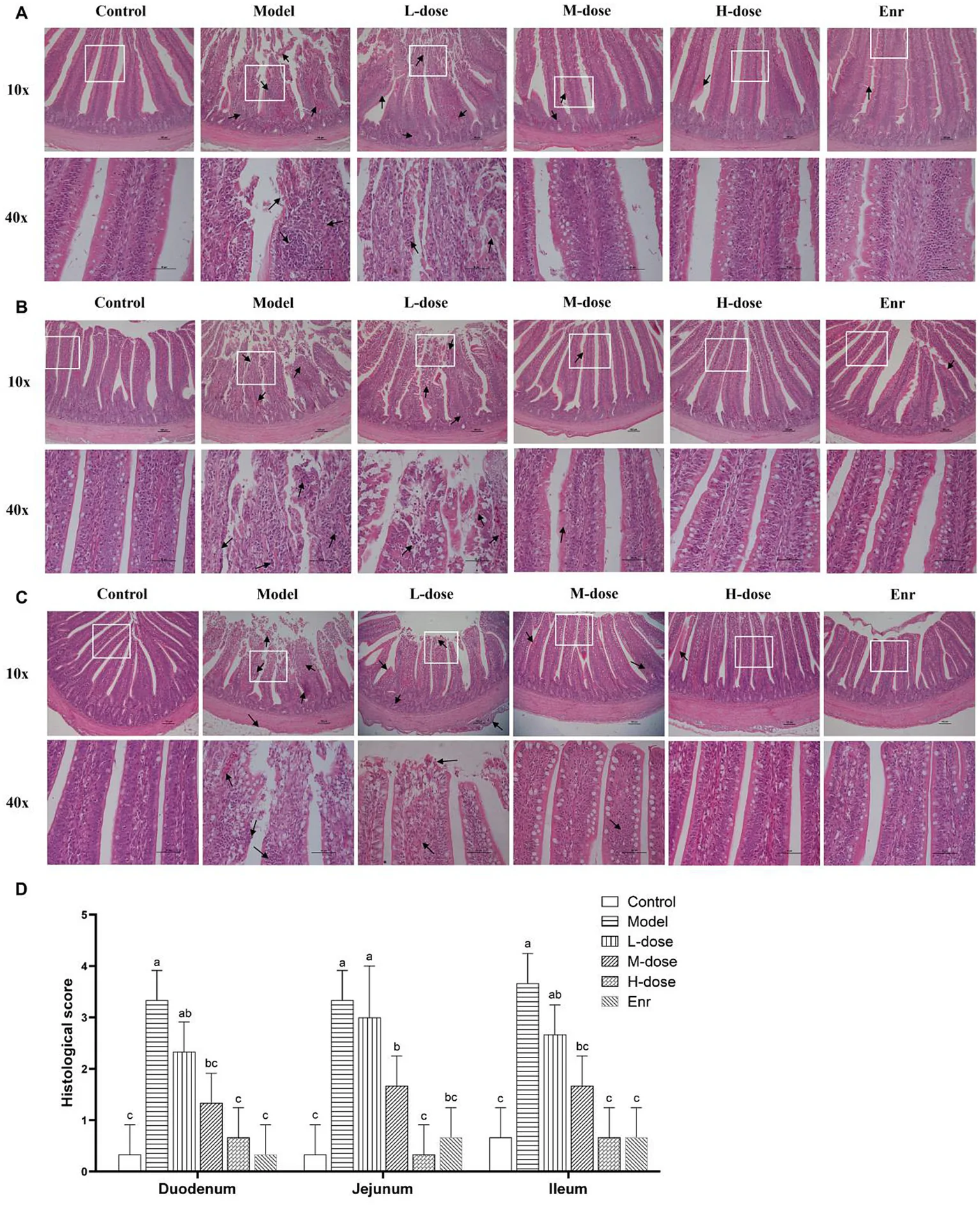
Repairing effects of QP on histopathology of *E. coli*-infected intestinal tissue in chicks. Histopathological changes in **(A)** duodenum, **(B)** jejunum, and **(C)** ileum (HE staining, original magnification: 100 × and 400×). **(D)** Histological score of duodenum, jejunum and ileum sections. Data are from at least three independent replicates and are expressed as mean ± SEM. The use of distinct lowercase letters marks significant between‑group differences (*p* < 0.05).

### 3.6 Impact of QP on expression of pyroptosis-related genes in organs of chicks infected with *E. coli*

To investigate the effect of QP on the pyroptosis pathway in major organs of *E. coli*-infected chicks, we employed qRT-PCR to detect mRNA expression levels of key pyroptosis pathway genes in liver and lung tissues. As shown in Figure 5, compared with the control group, the mRNA expression levels of NLRP3, GSDMD, Caspase-1, IL-1β, ASC, IL-18, and TNF-α were significantly upregulated (*p* < 0.05) in the liver and lung tissues of infected chicks, indicating a strong pyroptotic response in the organ tissues. Following drug intervention, all prophylactic QP pretreatment groups and the enrofloxacin positive control group significantly reversed the abnormal overexpression of these genes (*p* < 0.05). Notably, the prophylactic H-dose QP pretreatment group exhibited the most pronounced inhibitory effect, comparable to or superior than enrofloxacin, restoring most gene expressions to near-normal levels. These findings confirm at the genetic level that QP mitigates *E. coli*-induced inflammatory damage in chick organs by suppressing the expression of key genes in the pyroptosis signaling pathway.

**Figure 5:**
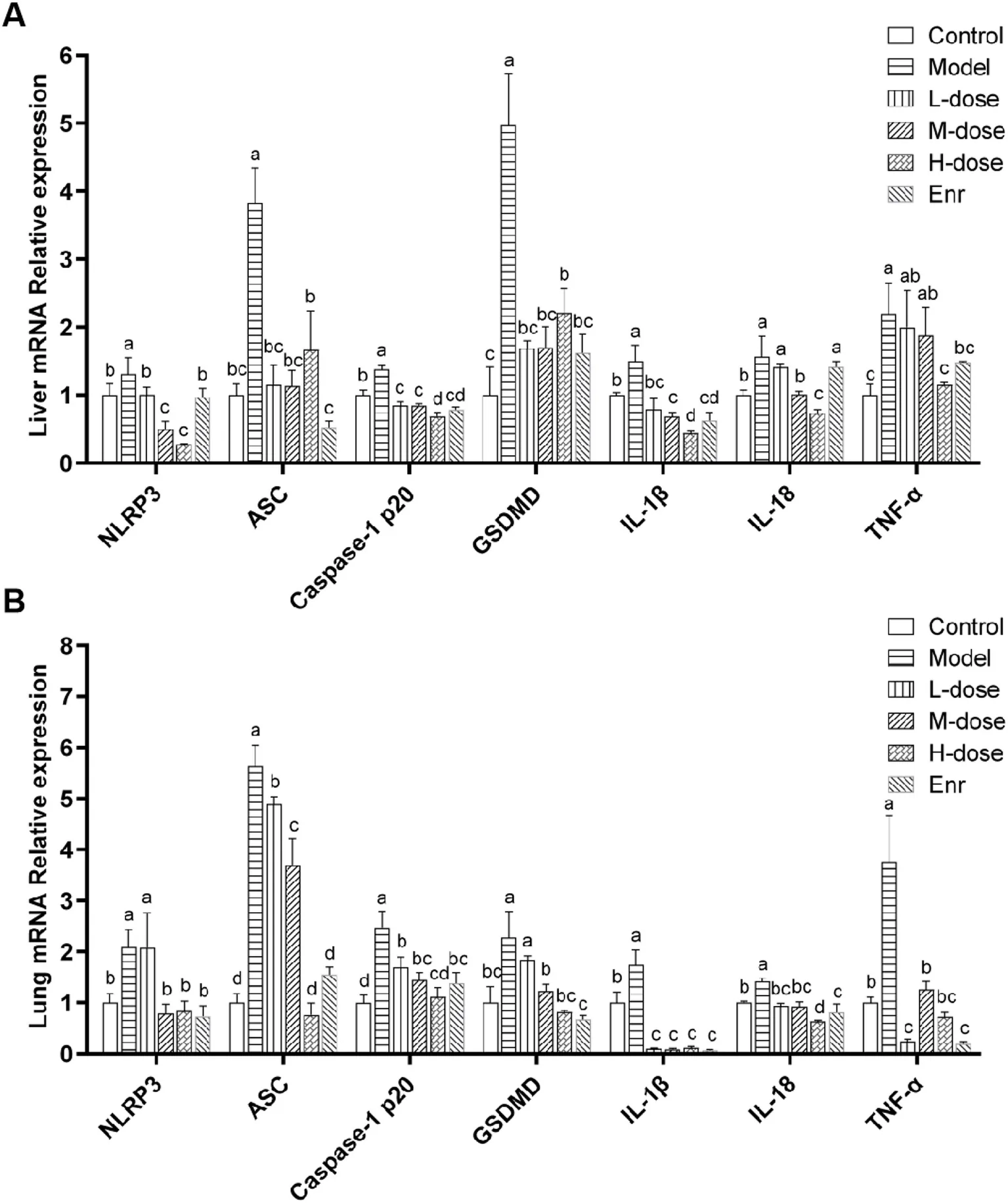
Impact of QP on expression of pyroptosis-related genes in organs of chicks infected with *E. coli*. qRT-PCR detection of mRNA expression levels of NLRP3, GSDMD, Caspase-1, IL-1β, ASC, IL-18, and TNF-α in **(A)** liver tissue and **(B)** lung tissue. Data are from at least three independent replicates and are presented as mean ± SEM. The use of distinct lowercase letters marks significant between‑group differences (*p* < 0.05).

### 3.7 Effect of QP on the expression levels of pyroptosis pathway genes in the intestines of *E. coli*-infected chicks

To investigate the effects of QP on the pyroptosis pathway in different intestinal segments, we further analyzed the expression of relevant genes in the duodenum, jejunum, and ileum of chicks. qRT-PCR results revealed that *E. coli* infection induced widespread activation of pyroptosis-related genes in the duodenum, jejunum, and ileum. Compared to the control group, mRNA expression levels of NLRP3, GSDMD, Caspase-1, IL-1β, ASC, IL-18, and TNF-α were significantly elevated (*p* < 0.05) in all infected intestinal segments (Figure 6). Following QP intervention, expression of all examined genes was significantly suppressed in a dose-dependent manner from the duodenum to the ileum (*p* < 0.05). These results clearly demonstrate that QP effectively suppresses the pyroptosis signaling pathway triggered by *E. coli* infection in the intestine (duodenum, jejunum, ileum).

**Figure 6:**
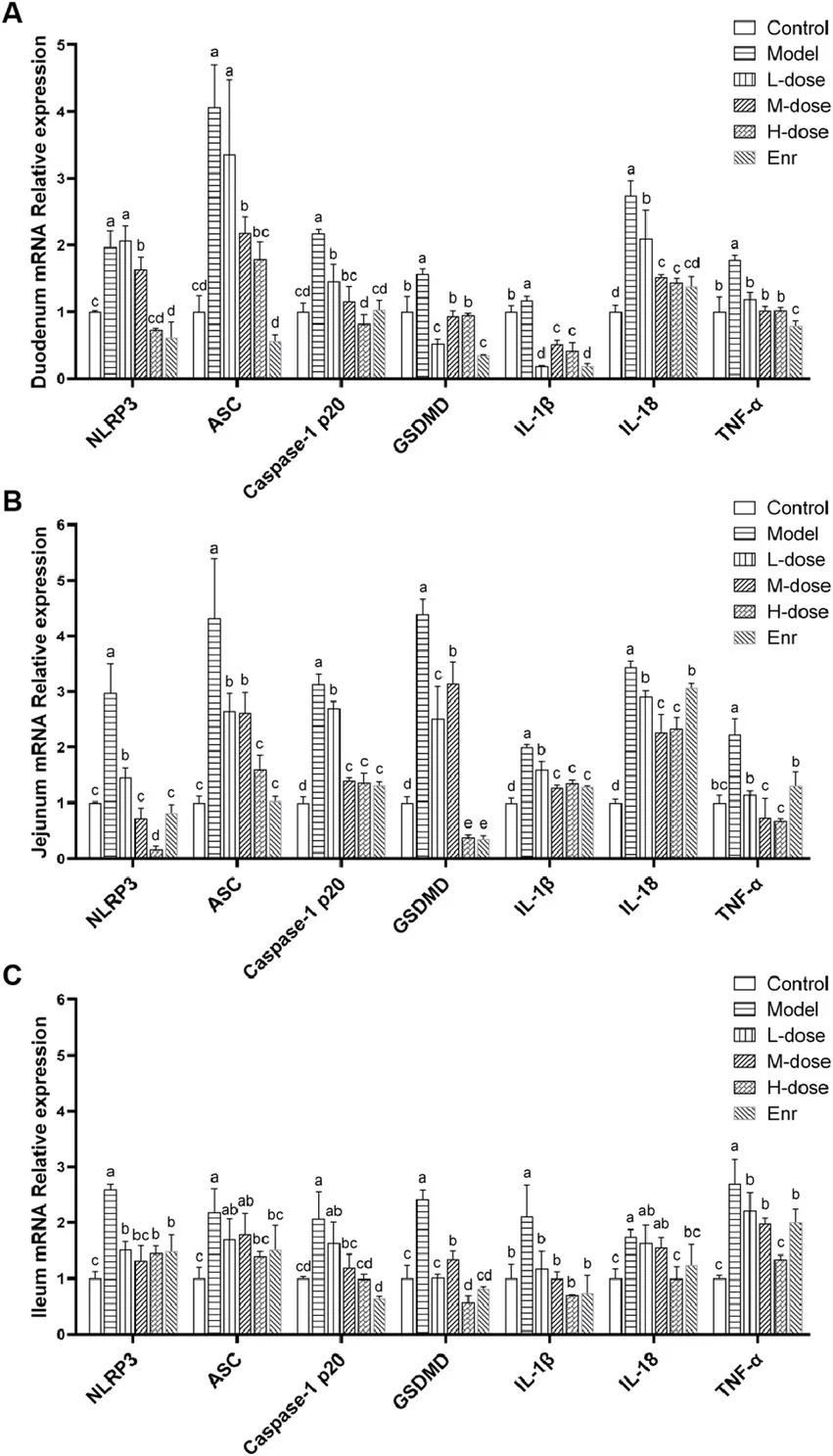
Effect of QP on the expression levels of pyroptosis pathway genes in the intestines of *E. coli*-infected chicks. qRT-PCR detection of mRNA expression levels of NLRP3, GSDMD, Caspase-1, IL-1β, ASC, IL-18, and TNF-α in **(A)** duodenum, **(B)** jejunum, and **(C)** ileum tissues. Data are from at least three independent replicates and are presented as mean ± SEM. The use of distinct lowercase letters marks significant between‑group differences (*p* < 0.05).

### 3.8 Effects of QP on pyroptosis pathway protein expression in organs of *E. coli*-infected chicks

To validate the regulatory role of QP at the protein level, we analyzed pyroptosis-related proteins in liver and lung tissues from chicks using Western blotting. Results (Figure 7) showed that consistent with gene-level changes, *E. coli* infection also triggered a robust pyroptotic response at the protein level: Compared with the control group, the model group exhibited significantly increased protein expression of NLRP3, GSDMD, Caspase-1 p20, IL-1β, and ASC in both liver and lung tissues (*p* < 0.05). Following drug intervention, all prophylactic QP pretreatment groups and enrofloxacin treatment significantly suppressed the abnormal expression of these key proteins (*p* < 0.05). The high-dose QP group exhibited particularly pronounced inhibitory effects. These Western blot data corroborated qRT-PCR results, confirming at the protein level that QP mitigates inflammatory damage to chick organs caused by *E. coli* by directly regulating key proteins in the pyroptosis pathway.

**Figure 7:**
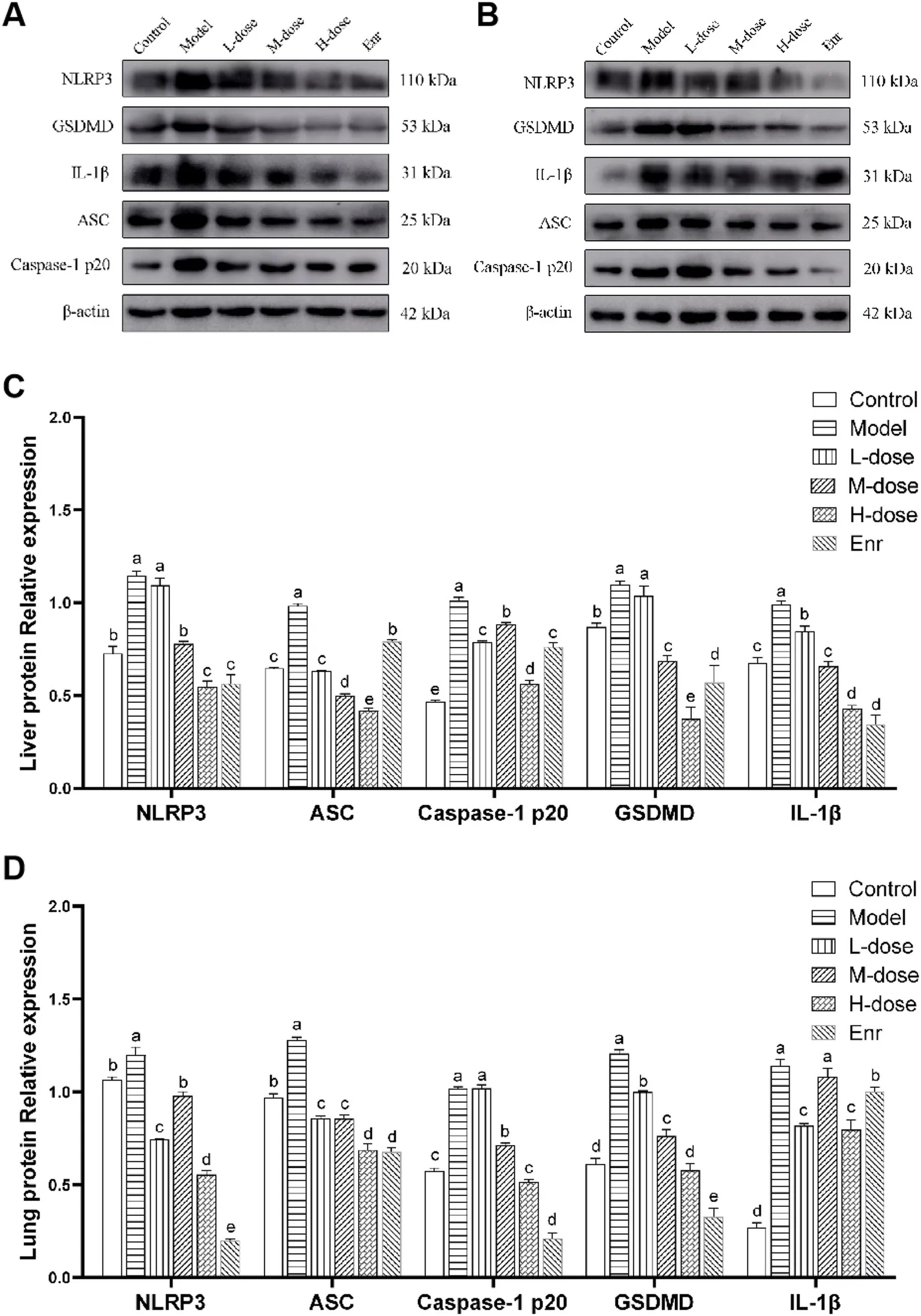
The effect of QP on the expression of organ necrosis related proteins in chickens infected with *E. coli*. Western blotting analysis of NLRP3, GSDMD, Caspase-1 p20, IL-1β, and ASC protein expression levels in **(A,C)** liver tissue and **(B,D)** lung tissue. Data are from at least three independent replicates and are presented as mean ± SEM. The use of distinct lowercase letters marks significant between‑group differences (*p* < 0.05).

### 3.9 Impact of QP on intestinal pyroptosis pathway protein expression in *E. coli*-infected chicks

We further systematically evaluated the effects of QP on the pyroptosis pathway in different intestinal segments at the protein level through Western blotting analysis of duodenal, jejunal, and ileal tissues. Figure 8 demonstrates that across the duodenum, jejunum, and ileum, protein expression of NLRP3, GSDMD, Caspase-1 p20, IL-1β, and ASC was significantly elevated in the model group relative to the control group (*p* < 0.05). Following QP intervention, these abnormally elevated pyroptosis-related proteins were significantly suppressed in a dose-dependent manner (*p* < 0.05) across all intestinal segments, exhibiting a trend consistent with gene expression changes. The high-dose QP demonstrated the most pronounced inhibitory effect. These results validated the effects across different intestinal segments at the protein level, ultimately confirming that QP effectively suppresses the activation of the pyroptosis pathway induced by *E. coli* infection in the intestine (duodenum, jejunum, ileum).

**Figure 8:**
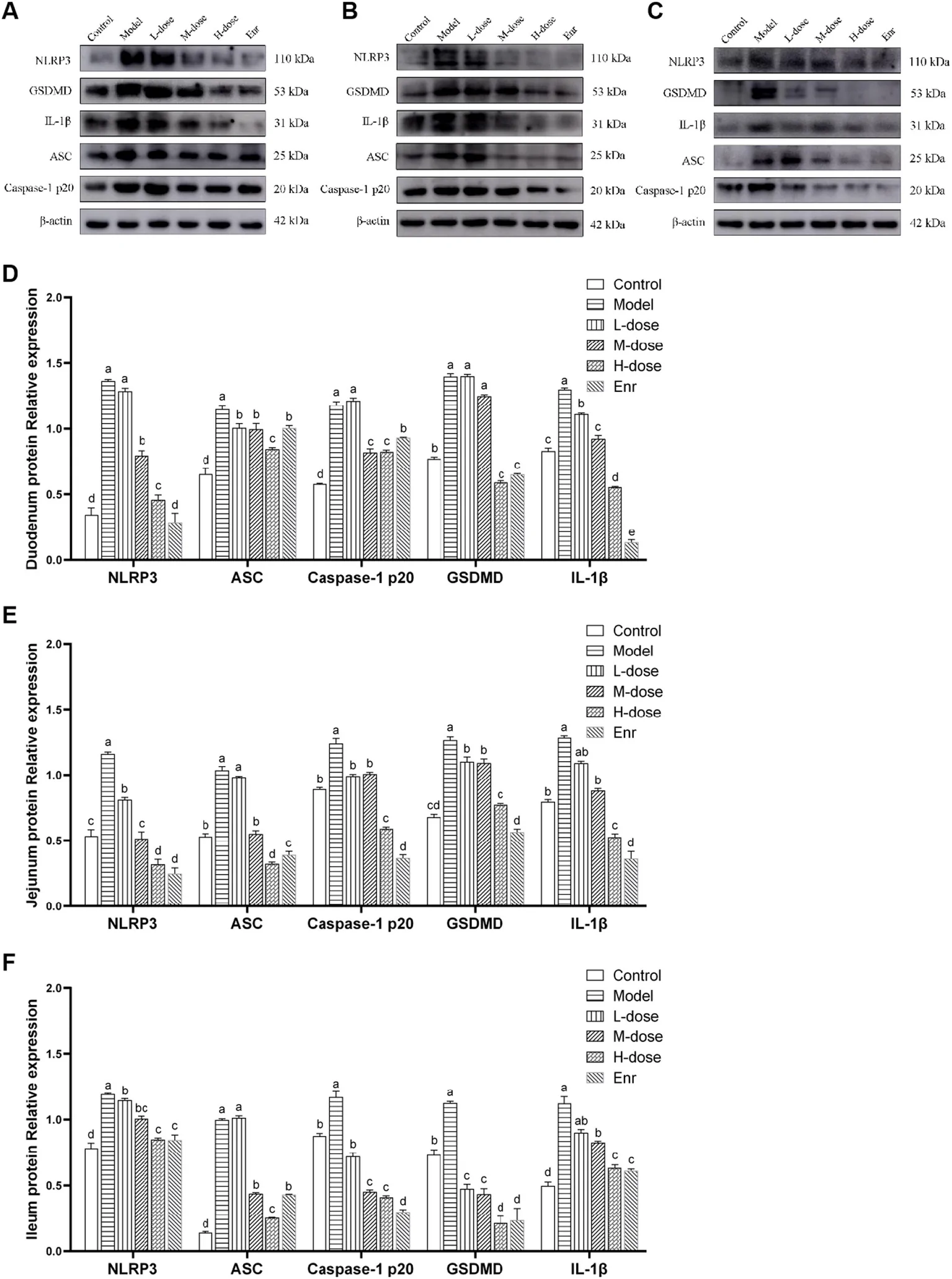
Impact of QP on intestinal pyroptosis pathway protein expression in *E. coli*-infected chicks. Western blotting analysis of NLRP3, GSDMD, Caspase-1 p20, IL-1β, and ASC protein expression levels in **(A,D)** duodenum, **(B,E)** jejunum, and **(C,F)** ileum tissues. Data are from at least three independent replicates and are presented as mean ± SEM. The use of distinct lowercase letters marks significant between‑group differences (*p* < 0.05).

### 3.10 Study on the effects of QP on LPS-induced enteritis in chicks

By establishing an LPS enteritis model in chicks whose gut microbiota was suppressed with antibiotics, we compared the therapeutic effects of QP treatment with and without gut microbiota to determine whether its efficacy depends on the presence of gut microbiota. Results indicated that chicks in the model group exhibited significantly elevated liver and spleen organ indices (*p* < 0.05) (Table 3), accompanied by slowed weight gain and markedly reduced thymus and bursa of Fabricius organ indices (*p* < 0.05) (Figure 9A). Compared with the model group, both the QP treatment group and the antibiotic depleted microbiota group improved the organ indices and weight abnormalities in chicks caused by LPS infection (*p* < 0.05), and no statistically significant difference was observed between the QP treatment group and the antibiotic depleted microbiota group. HE staining of ileal tissue revealed normal histological structure without obvious pathological changes in the control group chicks. While the model group exhibited marked intestinal mucosal structural damage, characterized by disrupted villus arrangement, epithelial cell detachment, loss of the lamina propria, and capillary congestion. In contrast, both the QP-treated and the antibiotic depleted microbiota groups showed significant repair of the intestinal mucosal structure, with no apparent pathological differences compared to the control group (Figures 9B,C). In summary, QP effectively alleviates LPS-induced enteritis in chicks by improving abnormal organ indices and repairing intestinal mucosal damage.

**Table 3:** Organ index analysis of LPS-induced inflammatory broilers.

Items (%)	Treatments				SEM	*P*-value
	Control	Model	QP	Antibiotic depleted microbiota		
Liver coefficient	24.83^b^	30.57^a^	22.65^c^	23.36^c^	0.561	0.001
Splenic coefficient	1.15^b^	2.19^a^	1.22^b^	1.17^b^	0.220	0.001
Bursae coefficient of Fabricius	5.05^a^	3.85^b^	4.54^ab^	4.73^a^	0.395	0.034
Thymus coefficient	6.99^a^	5.67^b^	6.96^a^	7.05^a^	0.540	0.044

Organ Index = Organ Weight (g)/Body Weight (kg). The use of distinct lowercase letters marks significant between-group differences (*p* < 0.05).

**Figure 9:**
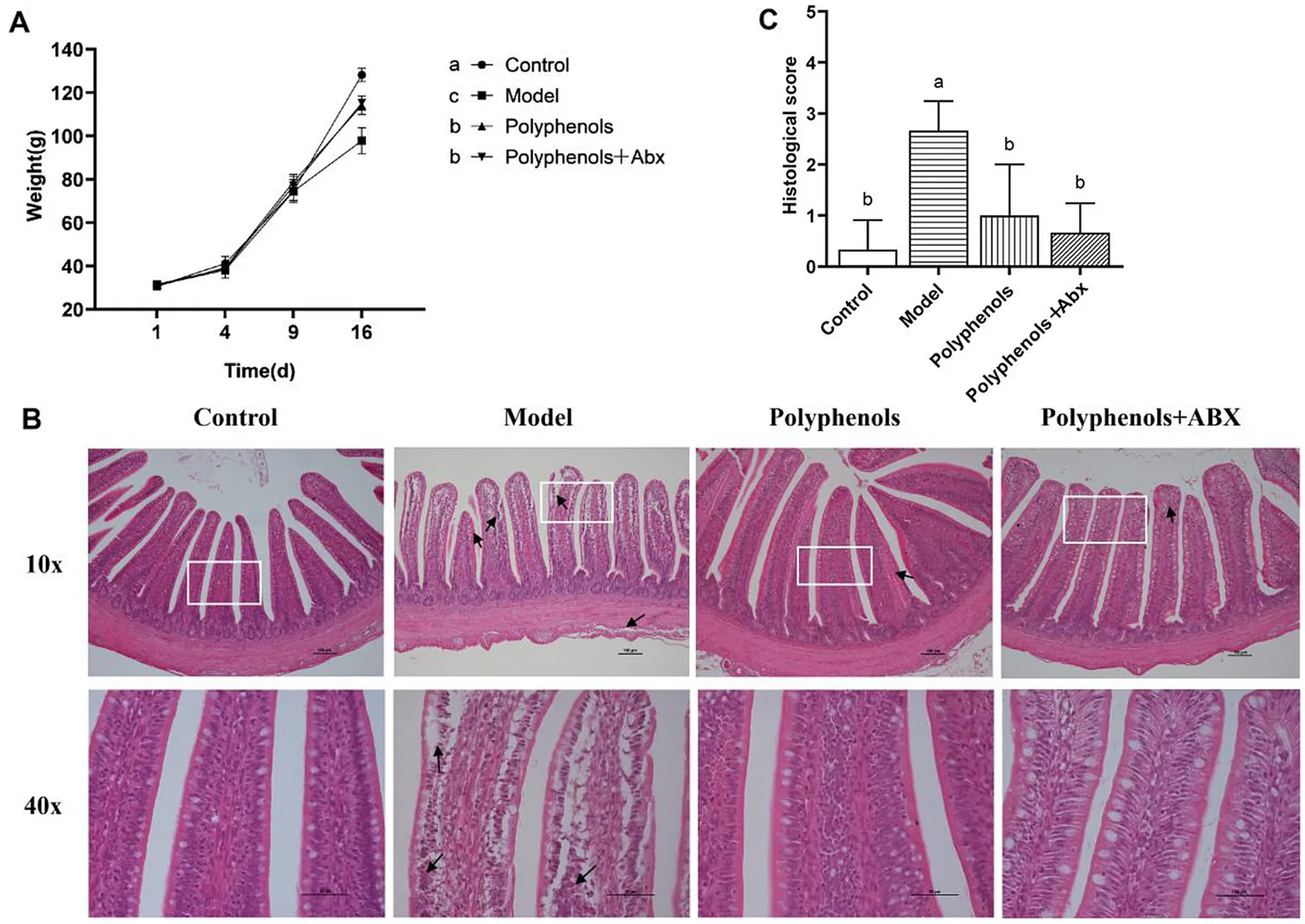
Effect of QP on LPS-induced enteritis in chicks. **(A)** Mean body weight changes in chicks across groups (*n* = 8). **(B)** Histopathological changes in ileal tissue (arrow indicates structural damage to the intestinal mucosa). HE staining; original magnification: ×100 and ×400. **(C)** Histological score of ileum sections. Data are from at least three independent replicates and are expressed as mean ± SEM. The use of distinct lowercase letters marks significant between‑group differences (*p* < 0.05).

### 3.11 Effects of QP on oxidative stress, MPO levels, and LDH release in chicks with enteritis

Subsequently, we measured oxidative stress markers, MPO levels, and LDH activity in the serum of chicks from each group. Results showed that compared with the control group, MDA levels in the serum of LPS-infected chicks were significantly elevated (*p* < 0.05) (Figure 10B), while SOD activity was significantly reduced (*p* < 0.05) (Figure 10A). Compared to the model group, both the QP-treated group and the antibiotic depleted microbiota treatment group significantly decreased MDA levels (*p* < 0.05) and increased SOD activity (*p* < 0.05). No statistically significant differences were observed between the QP-treated and antibiotic depleted microbiota treatment groups for these parameters. Regarding inflammatory responses, the model group exhibited significantly elevated serum MPO levels in chicks, indicating neutrophil infiltration (*p* < 0.05) (Figure 10C). Additionally, serum LDH activity was significantly increased in the model group (*p* < 0.05) (Figure 10D). Both the QP treatment group and the antibiotic depleted microbiota group effectively reversed the abnormalities in the aforementioned indicators, and no statistically significant differences were observed between the QP treatment group and the antibiotic depleted microbiota group in these indicators. This indicates that QP exerts equivalent therapeutic effects regardless of the presence or absence of gut microbiota.

**Figure 10:**
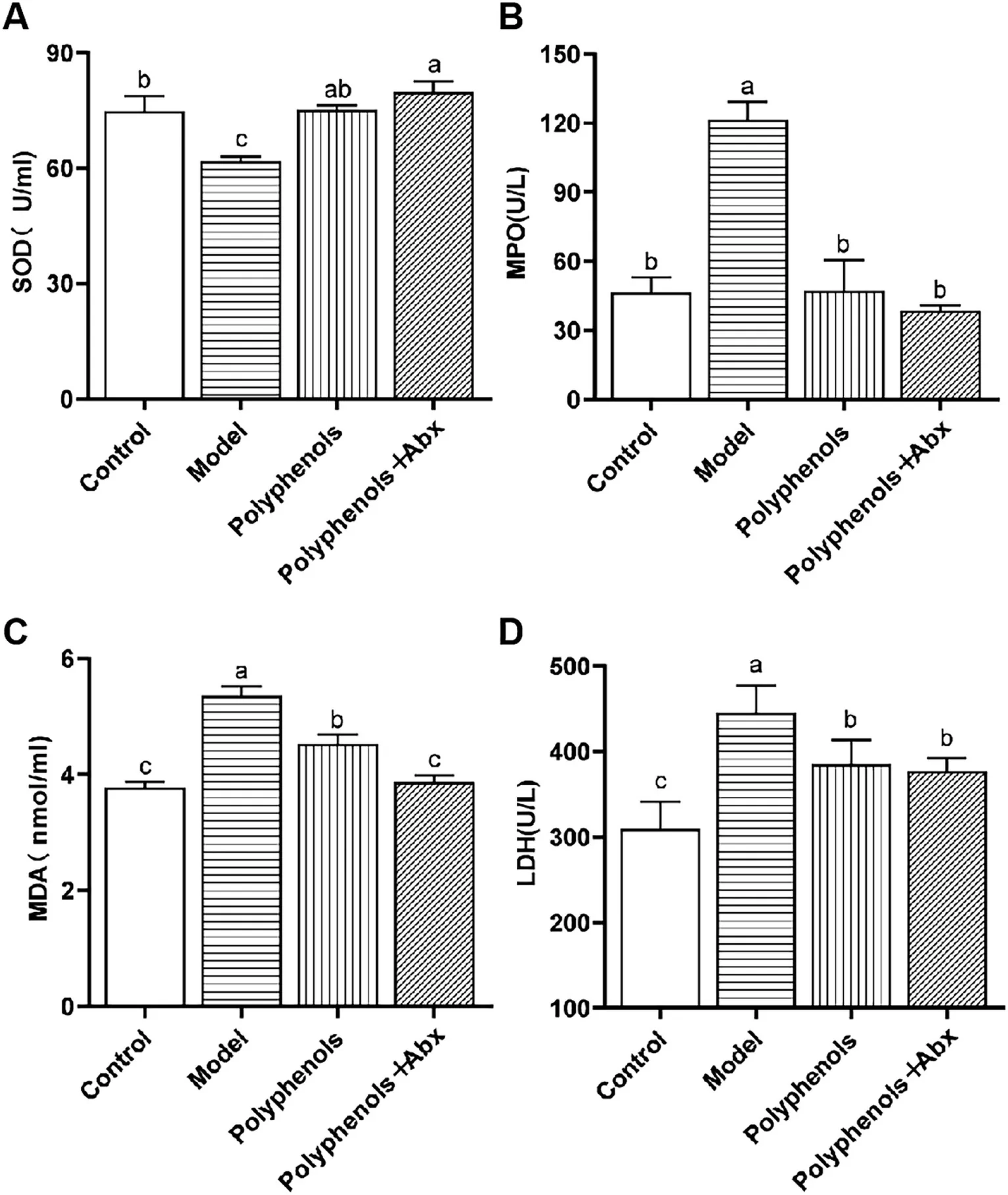
Effects of QP on oxidative stress, MPO Levels, and LDH release in chicks with enteritis. **(A)** Serum SOD activity. **(B)** Serum MDA content. **(C)** Serum MPO content. **(D)** Serum LDH release levels. Data are from at least three independent replicates and are expressed as mean ± SEM. The use of distinct lowercase letters marks significant between‑group differences (*p* < 0.05).

### 3.12 Investigating the mechanism of QP in alleviating intestinal inflammation in chicks

To investigate whether QP can still exert anti-inflammatory effects by inhibiting the pyroptosis pathway under antibiotic depleted microbiota conditions, Western blotting was used to detect the expression of key proteins in the pyroptosis pathway in the ileum tissue of chicks. Results showed that compared with the control group, the model group exhibited significantly increased protein expression of NLRP3, GSDMD, Caspase-1 p20, IL-1β, and ASC (*p* < 0.05) (Figures 11A,B). Compared with the model group, both the QP-treated group and the antibiotic depleted microbiota treatment group significantly reduced the expression levels of these proteins (*p* < 0.05). Notably, the antibiotic depleted microbiota treatment group exhibited significantly lower protein expression levels of NLRP3, IL-1β, and ASC than the QP-treated group (*p* < 0.05), while no significant differences were observed for the remaining proteins between the two groups. Further analysis of mRNA expression levels via qRT-PCR revealed that NLRP3, GSDMD, Caspase-1, IL-1β, ASC, IL-18, and TNF-α were significantly upregulated in the infection group (*p* < 0.05) (Figure 11C). The QP treatment group and the antibiotic depleted microbiota group treatment group significantly suppressed the overexpression of these genes (*p* < 0.05). Among them, the antibiotic depleted microbiota group treatment group showed significantly lower GSDMD and IL-1β gene expression levels compared to the QP treatment group (*p* < 0.05), while no significant differences were observed for the remaining genes between the two groups. These findings indicate that QP alleviates bacterial enteritis in chicks by modulating the host innate immune response and inhibiting the pyroptosis pathway. This protective effect may be independent of the gut microbiota and remains effective under antibiotic depleted microbiota group conditions. This protective effect may be independent of the gut microbiota and remains effective under antibiotic depleted microbiota group conditions.

**Figure 11:**
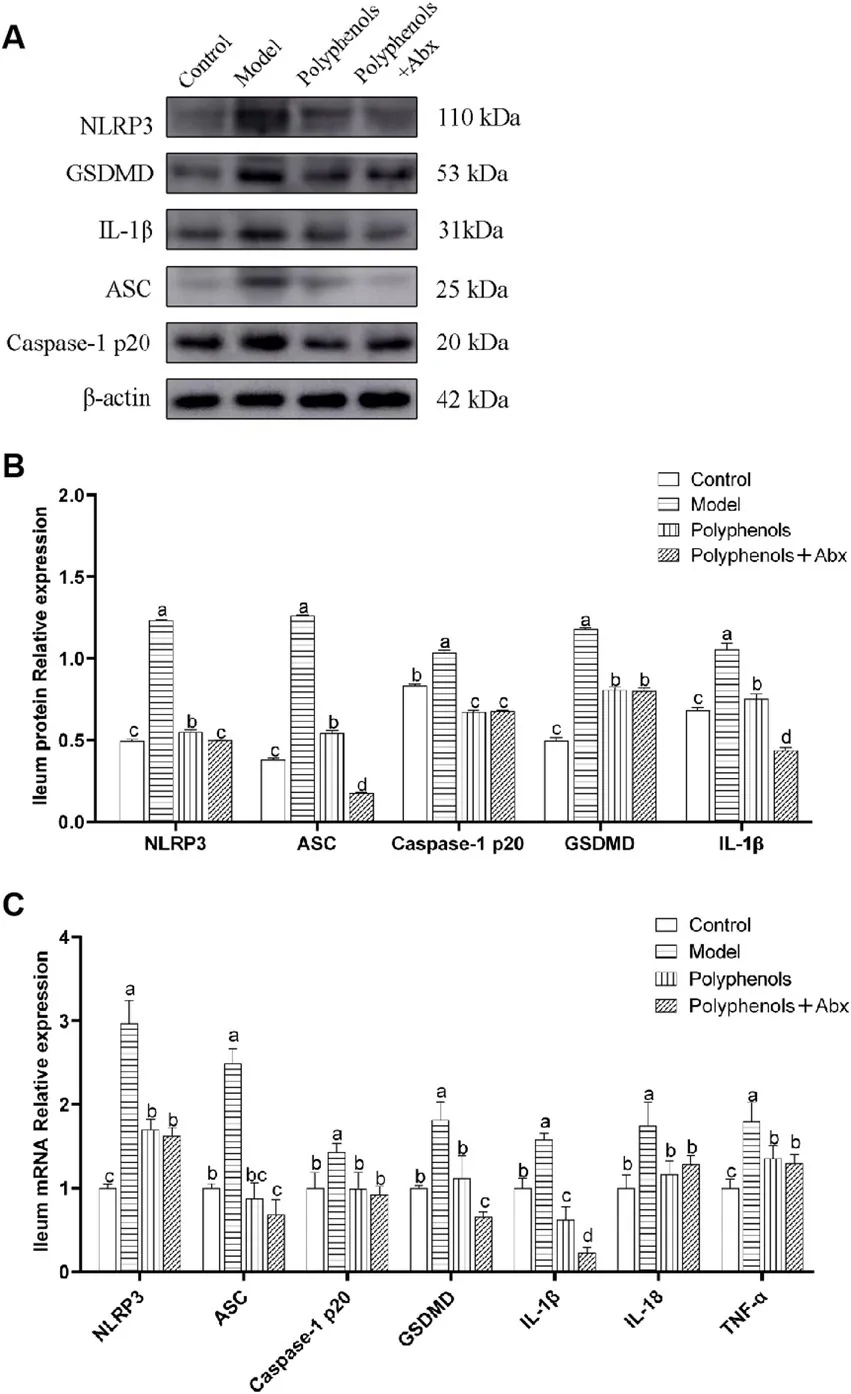
Investigating the mechanism of QP in alleviating intestinal inflammation in chicks. **(A,B)** Western blotting analysis of NLRP3, GSDMD, Caspase-1 p20, IL-1β, and ASC protein expression levels in ileum tissue. **(C)** qRT-PCR detection of mRNA expression levels of NLRP3, GSDMD, Caspase-1, IL-1β, ASC, IL-18, and TNF-α in ileum tissue. Data are from at least three independent replicates and are presented as mean ± SEM. The use of distinct lowercase letters marks significant between‑group differences (*p* < 0.05).

## 4 Discussion

Against the backdrop of increasingly severe antibiotic resistance, plant polyphenols have emerged as ideal candidates for antibiotic alternatives due to their multifunctionality, low resistance induction potential, and diverse pharmacological activities (13, 14). Multiple studies on natural polyphenol extracts have confirmed that pomegranate polyphenols exert antibacterial effects by differentially disrupting the cellular structure of Gram-negative bacteria (24); natural polyphenols extracted from Albariño white grape marc demonstrate potent inhibitory effects against clinically isolated *E. coli* strains (25); while polyphenolic components (such as lignans and phenylethanoid glycosides) from the fruit of the traditional Chinese medicine *Forsythia suspensa* have also been demonstrated to possess anti-*E. coli* activity (26). Polyphenol-based colloidal systems developed in recent years (e.g., chestnut and pine polyphenols) exhibit potent inhibitory effects against common drug-resistant bacteria in livestock farming (27). *In vitro* antimicrobial tests demonstrate that QP possesses direct bacteriostatic and bactericidal activity against *E. coli* (MIC and MBC both at 43.75 mg/mL). The high MIC value *in vitro* indicates that QP has weak direct bactericidal activity, and its protective effect *in vivo* is not primarily mediated by direct antibacterial activity. Instead, the active components of QP are absorbed into the body and target host immune cells, where they significantly inhibit overactivation of the pyroptosis pathway and reduce the release of pro-inflammatory factors.

QP exerts multi-organ protective functions through its potent anti-inflammatory and antioxidant effects, consistent with the characteristics of polyphenolic compounds. According to reports, Forsythiaside A ameliorates pulmonary inflammation and epithelial barrier disruption by co-activating the Nrf2/PPAR-γ pathway and suppressing the NF-κB pathway (28, 29). The primary tea polyphenol EGCG enhances broiler liver antioxidant capacity and intestinal barrier function while improving growth performance (30, 31). Plant polyphenols such as resveratrol and tannic acid, when used as feed additives, mitigate AFB1-induced growth suppression and organ damage in broilers through antioxidant and anti-inflammatory effects (32, 33). Phillygenin exerts multi-targeted anti-inflammatory effects across disease models by synergistically inhibiting NF-κB and modulating multiple pathways including Nrf2/MAPK, significantly improving arthritis, liver fibrosis, and colitis (34–36). Schisandrin A modulates the gut-liver axis to enhance intestinal barrier function and microbial balance, thereby reducing hepatic inflammation and oxidative stress, thus synergistically treating avian *E. coli* disease (37). The results of this study indicate that QP treatment significantly reduced MDA levels in the serum of infected chicks, increased SOD activity, suppressed the elevation of serum MPO levels, and reduced LDH release. Concurrently, it effectively improved abnormal organ indices and decreased bacterial loads in the liver, lungs, and intestines. HE staining results revealed that QP treatment also mitigated histopathological damage in the liver, lungs, and intestines (duodenum, jejunum, ileum). These findings indicate that QP can alleviate systemic oxidative stress and inflammatory responses, demonstrating multi-organ and systemic protective effects.

Research indicates that pyroptosis triggered by NLRP3 inflammasome activation is a key driver of multiple diseases, with its excessive activation playing a pivotal role in bacterial infection-related disorders (7, 12, 38). In various multi-organ injury models, plant polyphenols demonstrate significant protective effects by intervening in key signaling pathways of pyroptosis. For instance, dihydromyricetin significantly alleviates LPS-induced liver injury in chickens, a mechanism closely associated with pyroptosis inhibition (4). In the gut, both dihydromyricetin and quercetin alleviate LPS-induced intestinal inflammation, pyroptosis, and barrier dysfunction (39, 40). In the lungs, polyphenolic compounds such as echinacea polyphenols and Qingwen Hufei Granules have been demonstrated to mitigate LPS-induced acute lung injury by regulating pyroptosis-related pathways (41, 42). These studies consistently indicate that inhibiting pyroptosis represents a common key mechanism by which plant polyphenols counteract *E. coli* LPS-induced multi-organ damage. This study further confirmed at the gene and protein expression levels that QP significantly suppressed the expression of NLRP3, ASC, Caspase-1, Caspase-1 p20, GSDMD, IL-1β, and IL-18 in the liver, lung, and intestinal tissues (duodenum, jejunum, ileum) of *E. coli*-infected chicks. These preliminary results indicate that QP has a potential protective effect against the systemic injury induced by this experimental avian pathogenic *E. coli* challenge.

Currently widely used microbiota-dependent strategies (such as probiotics) show some efficacy, but their stability is easily disrupted by factors including microbiota composition, animal age, and environmental conditions (43, 44). Even non-antibiotic interventions (such as vaccination) can disturb gut microbiota structure (45). While diverse microbiota can provide colonization resistance through mechanisms like “nutritional blockade,” they remain dependent on the microbiome (46).

This study demonstrated in an antibiotic depleted microbiota group chick model that QP significantly alleviates LPS-induced intestinal inflammation and oxidative stress while effectively inhibiting pyroptosis. Its efficacy was comparable to that of the conventional QP group, and its inhibitory effects on key pyroptosis-related proteins such as NLRP3, IL-1β, and ASC were significantly pronounced under antibiotic depleted microbiota group conditions. Reports have shown that Polygonatum cyrtonema Hua polysaccharide alleviates ulcerative colitis by regulating inflammatory and immune responses independently of gut microbiota (47). Similarly, QP exerts a protective effect by inhibiting pyroptosis. Its efficacy in alleviating chick enteritis may not depend on gut microbiota regulation, a unique advantage that enhances its application potential in complex farming environments.

## 5 Conclusion

This study confirms that QP significantly alleviates multi-organ (liver, lung, intestine) damage and inflammatory responses induced by *E. coli* infection in chicks. Further mechanistic studies using an LPS-induced inflammation model confirmed that the inhibitory effect of QP on intestinal pyroptosis does not depend on the presence of intact gut commensal microbiota, providing preliminary mechanistic insight into its *in vivo* anti-inflammatory activity. Subsequent investigations revealed that this effect may primarily be mediated by inhibition of the pyroptosis signaling pathway, thereby synergistically contributing to its anti-inflammatory, antioxidant, and multi-organ protective effects. In summary, QP alleviate systemic damage caused by *E. coli* infection in chickens through gut microbiota-independent regulation of the pyroptosis pathway. The preliminary findings reported here offer partial mechanistic references for subsequent in-depth exploration of the *in vivo* biological activities of QP polyphenols in avian inflammatory and infection models.

## Data Availability

The original contributions presented in the study are included in the article/Supplementary material, further inquiries can be directed to the corresponding authors.
