# Supplementary material for: Qingqiao polyphenols may alleviate multi-organ inflammatory injury caused by Escherichia coli infection in chickens by regulating pyroptosis in a gut microbiota-independent manner
Source: Front Vet Sci. 2026 Oct 29;13:1976802. doi: 10.3389/fvets.2026.1976802 (PMC13754736; doi:10.3389/fvets.2026.1976802)
Supplement: Supplementary file 1 [file Image_1.pdf]

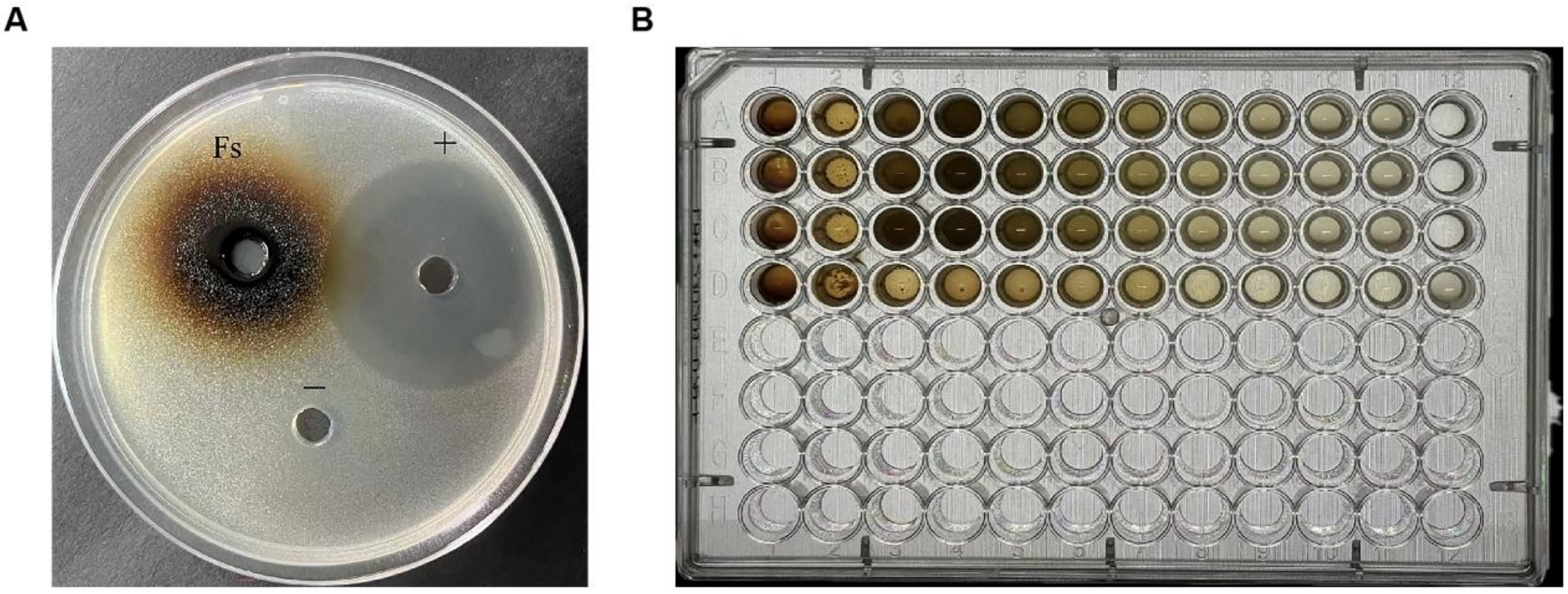

Figure S1. In vitro antibacterial activity of QP against *E. Coli*. (A) Drug susceptibility test results (“-” denotes negative control saline, “+” denotes positive control enrofloxacin, “Fs” denotes QP sample). (B) MIC determination of QP.
