# Supplementary material for: Qingqiao polyphenols may alleviate multi-organ inflammatory injury caused by Escherichia coli infection in chickens by regulating pyroptosis in a gut microbiota-independent manner
Source: Front Vet Sci. 2026 Oct 29;13:1976802. doi: 10.3389/fvets.2026.1976802 (PMC13754736; doi:10.3389/fvets.2026.1976802)
Supplement: Supplementary file 2 [file Table_1.docx]

Table 1S Histopathological scores.

Score	liver	lung	Duodenum/jejunum/ileum
0 (normal)	The hepatic lobules and sinusoids appear normal, with no signs of congestion or inflammatory cells.	The structure of the pulmonary lobules is intact, the alveolar spaces are clearly defined, and there is no inflammation.	Villi are normal, the mucosa is intact, and there are no inflammatory cells.
1 (mild)	Mild disorganization of the hepatic lobules, slight congestion, and scattered inflammatory cells	Focal thickening of the alveolar walls with a small number of inflammatory cells	Slightly disorganized villi, mild thickening of the mucosa, and occasional inflammatory cells
2 (moderate)	Disruption of hepatic lobules, mild narrowing of hepatic sinusoids, moderate inflammatory cell infiltration, and swollen hepatocytes	Partial lobular destruction, mild stromal widening, moderate inflammatory cell infiltration	Disorganized villi with partial fragmentation, thickened mucosa, and moderate inflammatory infiltration
3 (severe)	Marked disruption of the hepatic lobules, marked narrowing of the hepatic sinusoids, widespread congestion, and a large number of inflammatory cells; hepatocytes are swollen.	Marked destruction of alveoli, marked thickening of the stroma, numerous inflammatory cells, and alveolar narrowing	Visible villous breaks or shedding, mucosal damage, numerous inflammatory cells, and vascular injury
4 (extremely severe)	Severe destruction of the hepatic lobules, obstruction of the hepatic sinusoids, severe congestion/hemorrhage, and numerous foci of inflammatory cells; hepatocyte necrosis	Severe destruction of alveoli, interstitial fusion, alveolar obstruction, and severe inflammatory cell infiltration with congestion	Severe loss of villi, severe damage to the mucosa, extensive inflammatory infiltration, structural fragmentation, and hemorrhage or necrosis
